# Navigating Infertility in Kartagener's Syndrome: A Clinical Case Report

**DOI:** 10.7759/cureus.58635

**Published:** 2024-04-20

**Authors:** Tanya Ratnani, Meet P Kachhadia, Ninia Goyal, Deepak Singla, Gurjot Singh, Piyush Puri, Yuvraj Kaushal

**Affiliations:** 1 Internal Medicine, Adesh Institute of Medical Science and Research, Bathinda, IND

**Keywords:** kartagener's syndrome, situs inversus with dextrocardia, azoospermia, inflammation and infertility, infertility

## Abstract

Kartagener's syndrome is a genetic condition that is caused by a triad of symptoms, which includes bronchiectasis, chronic sinusitis, and situs inversus, and is considered rare. It is caused by defective ciliary motility, leading to impaired mucociliary clearance. We report a case of a 24-year-old male who presented with primary infertility and a long-standing history of recurrent respiratory infections. Physical examination revealed dextrocardia and digital clubbing. Radiological investigations confirmed situs inversus totalis and bronchiectasis. Semen analysis revealed azoospermia. Genetic analysis was not done due to financial constraints. However, the constellation of clinical features was diagnostic of Kartagener's syndrome. The patient was managed with antibiotics and chest physiotherapy. In vitro fertilization (IVF) was advised for infertility. A successful pregnancy was achieved through IVF, indicating viable sperm despite immotility. The aforementioned case underscores the significance of maintaining a heightened sense of suspicion for Kartagener's syndrome in individuals exhibiting unexplained bronchiectasis and infertility. Early diagnosis can prevent chronic respiratory morbidity and enable parenthood through assisted reproduction.

## Introduction

Kartagener's syndrome (KS) was first reported by Siewert in 1904, and later, Kartagener recognized the clinical diagnosis in 1933. Kartagener's syndrome is a rare autosomal recessive disorder. The clinical triad of situs inversus, bronchiectasis, and chronic sinusitis makes up this condition [1]. Camner et al. initially proposed ciliary dyskinesia as the reason for KS in 1975. In 1977, Eliasson et al. referred to KS as "immotile cilia syndrome" to describe the combination of infertility and recurrent sinopulmonary infections [2, 3]. Sperm motility, appropriate visceral orientation throughout development, and respiratory host defense all depend on the normal ciliary activity. Mutations in DNAI1 and DNAH5 genes cause ciliary motility to be decreased in KS, which increases the risk of infertility, recurrent sinopulmonary infections, and problems in left-right body orientation [4, 5].

The following characteristics, in addition to a history of rhinitis and persistent bronchial infections throughout early childhood, are indicated as diagnostic criteria for this syndrome: (a) patients or their siblings with situs inversus or dextrocardia; (b) live but immotile spermatozoa; (c) no or reduced tracheobronchial clearance; and (d) cilia exhibiting a distinctive ultrastructural abnormality on electron microscopy [6, 1].

## Case presentation

A 24-year-old Indian male, born from a non-consanguineous marriage in a rural area, presented to the infertility clinic alongside his wife, citing concerns regarding primary infertility. The couple, married for two years, had been actively attempting to conceive for the past year without success. However, the patient's medical history extends back to his adolescence.

At the age of 14, he exhibited mild nasal congestion, initially perceived as normal allergic manifestations given his rural upbringing. Over time, these symptoms escalated. By age 18, he experienced recurrent nasal congestion, facial pain, pressure, and a productive cough. Despite undergoing multiple courses of antibiotics and decongestants, his symptoms persisted.

Furthermore, his medical dossier revealed grade 2 exertional dyspnea, particularly notable during activities such as cycling and climbing stairs, persisting for over two years. Additionally, he experienced several instances of copious greenish sputum production, foul-smelling cough, sporadic episodes of acute epistaxis, and irregular bowel habits within the past four to five months.

Despite the concerning nature of his symptoms, the patient did not undergo any fertility evaluations or comprehensive medical assessments due to the limited access to healthcare resources in his rural locale. His family history was unremarkable, with no significant medical conditions such as hypertension, diabetes mellitus, or chronic respiratory ailments.

The patient admitted to social smoking, consuming five to six cigarettes per week over seven years, which he subsequently discontinued upon commencing attempts to conceive with his wife. On physical examination, he appeared alert and oriented, with no overt abnormalities noted.

However, upon further examination, grade 2 digital clubbing and a right-sided apex beat in the fifth intercostal space were observed. Auscultation revealed bilateral wheezing and right basal crackles, with accentuated heart sounds predominantly localized to the right hemithorax.

These clinical findings strongly suggested the presence of Kartagener's syndrome, characterized by the triad of chronic sinusitis, bronchiectasis, and situs inversus. The patient was initiated on conservative management comprising mucolytics, prophylactic antibiotics, inhaled antimuscarinic bronchodilators, and steroid nasal sprays, resulting in a favorable response.

Given the intricate nature of his condition and the absence of comprehensive medical facilities in his rural setting, the diagnosis and treatment of his condition were significantly delayed until his presentation at the infertility clinic prompted further evaluation.

In light of the diagnosis and the couple's fertility concerns, the infertility clinic recommended considering alternative conception methods such as in vitro fertilization or adoption.

Table 1 shows the results of a complete blood count panel. His serum levels of sex hormones and thyroid hormones fell within the established normal ranges. Additionally, screening for viral markers, encompassing human immunodeficiency virus (HIV), hepatitis B surface antigen (HBsAg), and hepatitis C (HCV), yielded negative results. Furthermore, analysis of his sputum demonstrated the absence of acid-fast bacilli (AFB). 

**Table 1:** Complete blood count

Parameter	Result	Normal values
White blood cell (WBC) count	14,000 cells/µL	4500-11,000 cells/µL
Red blood cell (RBC) count	4.7 million/µL	4.35-5.65 million/µL
Hemoglobin (Hb or Hgb)	15 g/dL	14-18 g/dL
Hematocrit (Hct)	42.4%	40-54%
Mean corpuscular volume (MCV)	85 fL	80-100 fL
Mean corpuscular hemoglobin (MCH)	31 pg	27-31 pg
Mean corpuscular hemoglobin concentration (MCHC)	32 g/dL	32-36 g/dL
Platelet count	250,000/µL	150,000-400,000/µL

**Figure 1:**
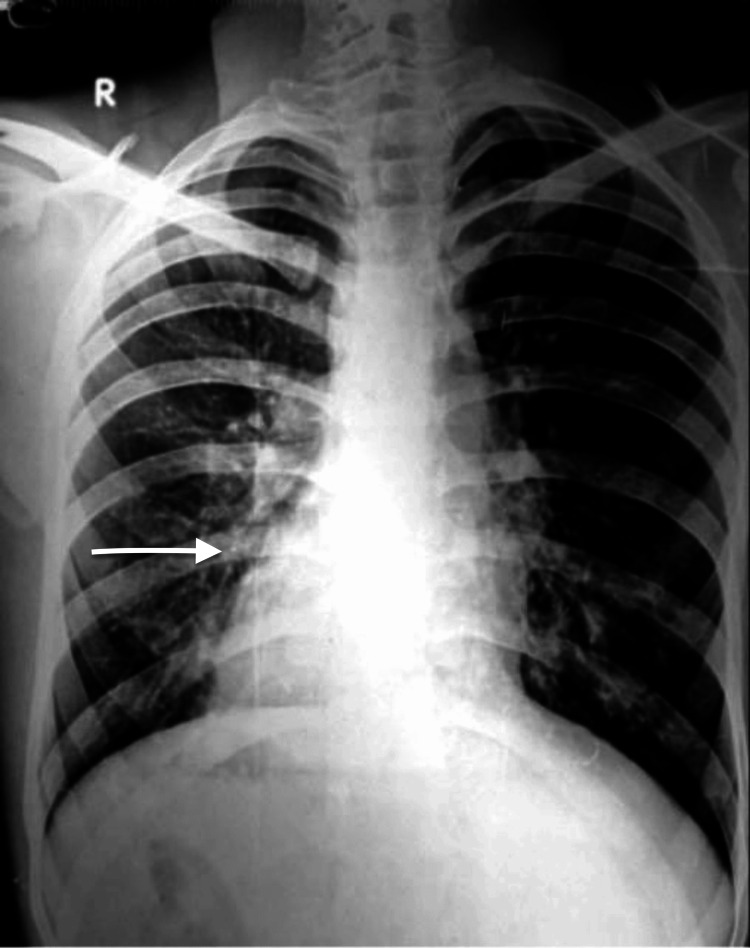
Chest X-ray revealing dextrocardia

A routine chest X-ray was performed, revealing an incidental observation of dextrocardia (Figure 1).

**Figure 2:**
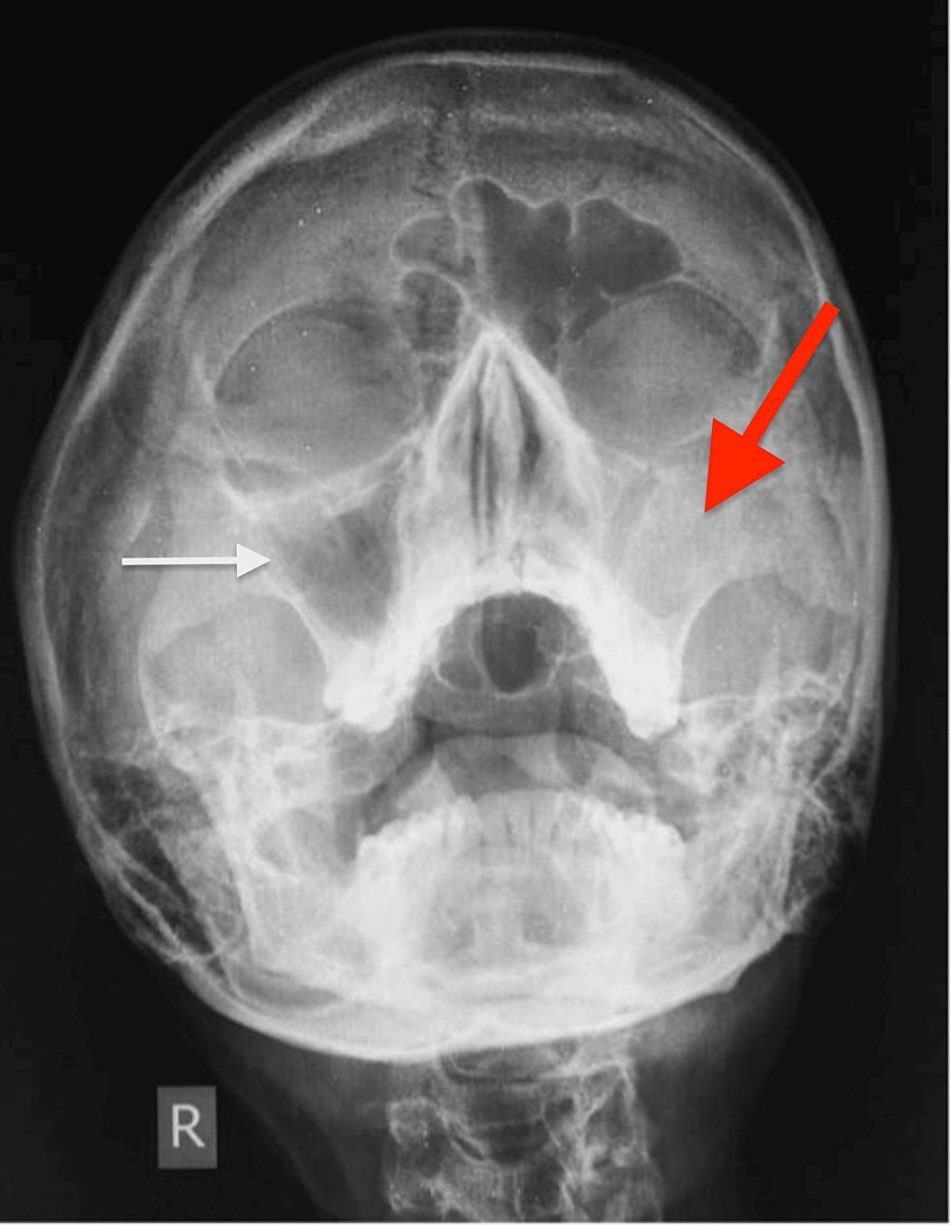
Water's view Maxillary sinusitis is evident more on the left side than on the right side.

The water's view or the occipito-mental X-ray of his paranasal sinuses showed mild opacities in the right maxillary sinus which indicated sinusitis (Figure 2).

**Figure 3:**
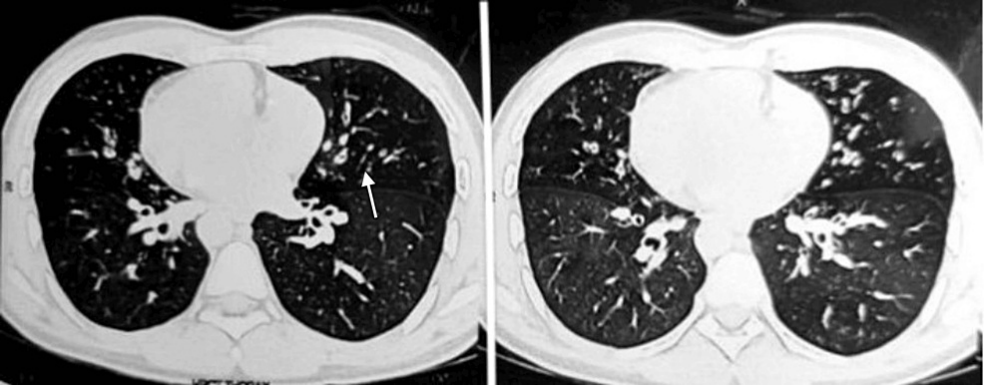
HRCT High-resolution computed tomography (HRCT) scan of the chest, revealing multiple cavitary lesions having a "signet ring" appearance and a "tram-track" sign due to bronchial wall thickening suggestive of bronchiectasis.

The patient was further sent for a high-resolution chest CT (HRCT) and it revealed multiple discrete and confluent nodules showing the tree-in-bud pattern in bilateral lung fields with peribronchial wall thickening suggestive of an infectious etiology likely to be bronchiolitis or bronchiectasis (Figure 3).

**Figure 4:**
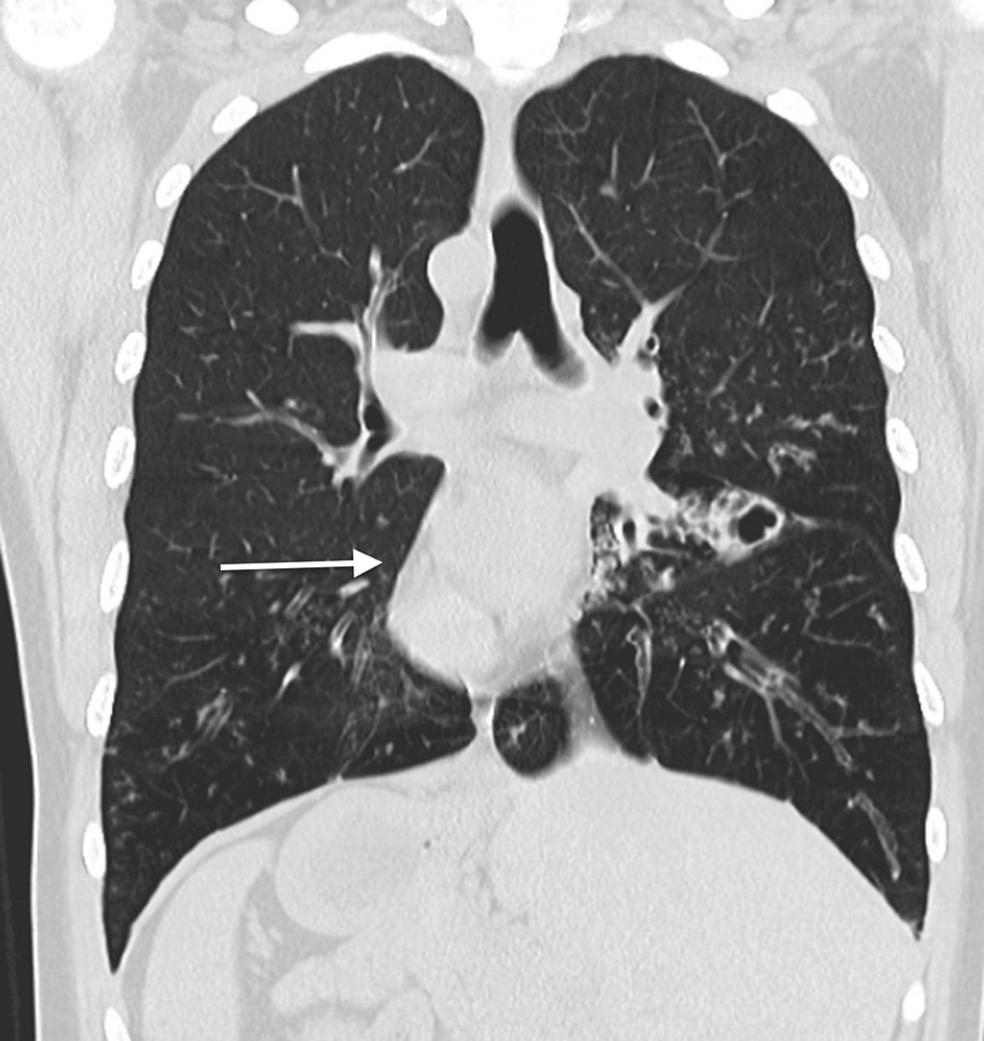
High-resolution computed tomography (HRCT) High-resolution computed tomography (HRCT) scan of thorax revealing bronchial wall thickening, cystic cavitary lesions in bilateral lungs and dextrocardia. Furthermore, the liver is seen on the right side and spleen on the left, suggesting situs inversus totalis.

The HRCT also revealed an incidental finding of dextrocardia with the liver on the left side and spleen on the right side s/o situs inversus totalis, supporting a strong association with the dextrocardia visible in the chest X-ray (Figure 4).

A provisional diagnosis encompassing primary ciliary dyskinesia or Kartagener's syndrome was postulated. To further elucidate the infertility concerns, seminal analysis was conducted, and his spouse underwent an array of investigations including hysterosalpingography to assess tubal patency, evaluation of thyroid hormone levels, anti-mullerian hormone levels, and ovarian reserve count. Notably, all assessments pertaining to female fertility factors yielded results falling within the established normal parameters. Conversely, the seminal analysis revealed normative values for viscosity, liquefaction, and pH. However, a profound absence of sperm motility and azoospermia were unequivocally documented, as illustrated in Table 2.

**Table 2:** Sperm analysis HPF - high power field; NF - normal form; RBC - red blood cell

Semen parameter	Result	Normal range
Duration of abstinence	2-7 days	-
Ejaculation-analysis interval	60 minutes	-
Liquefaction	Complete	-
Appearance	Normal	-
Viscosity	Normal	-
Volume	≥1.5 mL	≥ 1.5 mL
pH	≥7.2	7.2-7.8
Sperm count	0 sperm/mL	39 million per ejaculate
Motility	N/A (no sperm to assess)	40% or more
Morphology	N/A (no sperm to assess)	4-14% NF
Pus cell	3-5/HPF	1-2/HPF
RBC count	NIL/HPF	NIL/HPF

All the above history gave a strong indication of the triad of Kartagener's syndrome, i.e., chronic sinusitis, bronchiolitis, and situs inversus. The patient was put on conservative management of mucolytics, prophylactic antibiotics, inhaled antimuscarinic bronchodilators, and steroid sprays which he responded quite well to. The infertility clinic advised the couple to consider other methods of conception like invitro-fertilization and adoption.

The couple agreed to go for an in vitro fertilization. In an outpatient follow-up after four months, no new respiratory symptoms were reported.

## Discussion

Immotile-cilia syndrome is an uncommon genetic condition that is inherited autosomally recessively and results in malfunctioning of the cilia in the respiratory tract and fallopian tube [7]. It causes otitis media, pneumonia, bronchiectasis, and chronic rhinosinusitis, frequently as a result of pseudomonal infection [8]. Different symptoms are present in Kartagener's syndrome, which is characterized by situs inversus, chronic sinusitis, and bronchiectasis [9].

The motile cilia in the testicular efferent ductules of males with Kartagener's syndrome may be similarly dyskinetic to the cilia in the respiratory tract. Sperm may clump together if ciliary motion in the testis's efferent ductules is compromised in primary ciliary dyskinesia (PCD). This might result in decreased motility, decreased sperm survival, and an inability for the sperm to pass through the female reproductive canal and fertilize [10].

Further support for this notion is provided by testicular biopsies, which show reduced numbers of ciliated cells in the efferent ductule, sperm agglutination, and ductal blockage; in another such case with Kartagener's syndrome, the cilia in the efferent ductule were found to be absent [11]. After the Tru-Cut biopsy (Merit Medical, South Jordan, Utah), there were no hematomas or scars, anti-sperm antibodies did not form, and there was no increase in pituitary gonadotropins or decrease in testosterone levels [12].

Tests for compromised cilia function, biopsies, and genetic research are also part of the diagnosis. The measurement of exhaled nasal nitric oxide has a strong negative predictive value, making it a useful screening technique [13]. Inhaled colloid albumin tagged with 99Tc can be used to assess the impaired mucociliary transport observed in these patients [14]. Abnormalities in the structure of cilia are shown by electron microscopy of biopsy samples [15].

It is reported that patients with specific ciliary ultrastructural defects seen by electron microscopy, namely "inner dynein arms with microtubular disorganization" and "no inner and outer dynein arms." are more likely to be associated with infertility [16]. DNAI1 and DNAH5 mutations can be tested genetically; biallelic mutations support the diagnosis, whereas a single allelic mutation does not.

Antibiotics with pseudomonal coverage, supportive pulmonary care, and daily physical therapy for the chest are the three mainstays of treatment for Kartagener's syndrome. Acetylcysteine, DNase, and hypertonic saline may be considered in individuals with recurrent infections or prolonged respiratory symptoms; however, their efficacy is unknown. In extreme cases, postponing a bilateral lung transplant could have disastrous consequences [17].

Immotile-cilia syndrome differential diagnosis includes malignancy, interstitial lung illnesses (idiopathic pulmonary fibrosis, idiopathic interstitial pneumonia), and disorders associated with bronchiectasis. Inherited reasons (foreign body aspiration, tumors, lymphadenopathy, chronic obstructive lung disease, mucoid impaction) and acquired causes (bronchomalacia, pulmonary sequestration, yellow nail syndrome) are among them. The differential diagnosis also includes other illnesses (alpha-1), abnormal secretory disorders (cystic fibrosis), and recurring infections (immunodeficiencies).

## Conclusions

This case highlights the classical presentation of Kartagener's syndrome with the triad of situs inversus totalis, chronic sinusitis, and bronchiectasis in a young male with infertility. The impaired ciliary motility affecting the respiratory tract and reproductive system was confirmed with imaging and semen analysis. Early diagnosis and management with medications for sinusitis and lung infections can improve the quality of life in these patients. However, infertility remains a challenge that may require assisted reproductive techniques like IVF, as successfully implemented in this patient. A multidisciplinary approach involving pulmonology, ENT, cardiology, gastroenterology, and reproductive medicine is crucial for the optimal management of the various manifestations of this rare genetic disorder. Increased awareness among clinicians regarding this syndrome can help in prompt diagnosis and better patient outcomes.
